# Serotonin Deficiency Increases Context-Dependent Fear Learning Through Modulation of Hippocampal Activity

**DOI:** 10.3389/fnins.2019.00245

**Published:** 2019-04-24

**Authors:** Jonas Waider, Sandy Popp, Boris Mlinar, Alberto Montalbano, Francesco Bonfiglio, Benjamin Aboagye, Elisabeth Thuy, Raphael Kern, Christopher Thiel, Naozumi Araragi, Evgeniy Svirin, Angelika G. Schmitt-Böhrer, Renato Corradetti, Christopher A. Lowry, Klaus-Peter Lesch

**Affiliations:** ^1^Division of Molecular Psychiatry, Center of Mental Health, University of Würzburg, Würzburg, Germany; ^2^Department of Neuroscience, Psychology, Drug Research, and Child Health, University of Florence, Florence, Italy; ^3^Max Delbrück Center for Molecular Medicine in the Helmholtz Association, Berlin, Germany; ^4^Charité – Universitätsmedizin Berlin, Berlin, Germany; ^5^Laboratory of Psychiatric Neurobiology, Institute of Molecular Medicine, I.M. Sechenov First Moscow State Medical University, Moscow, Russia; ^6^Department of Psychiatry, Psychosomatics, and Psychotherapy, Center of Mental Health, University of Würzburg, Würzburg, Germany; ^7^Department of Integrative Physiology and Center for Neuroscience, University of Colorado Boulder, Boulder, CO, United States; ^8^Department of Translational Psychiatry, School for Mental Health and Neuroscience, Maastricht University, Maastricht, Netherlands

**Keywords:** tryptophan hydroxylase 2, knockout, fear learning, extinction, long-term potentiation, hippocampus, immediate-early gene, serotonin deficiency

## Abstract

Brain serotonin (5-hydroxytryptamine, 5-HT) system dysfunction is implicated in exaggerated fear responses triggering various anxiety-, stress-, and trauma-related disorders. However, the underlying mechanisms are not well understood. Here, we investigated the impact of constitutively inactivated 5-HT synthesis on context-dependent fear learning and extinction using tryptophan hydroxylase 2 (*Tph2*) knockout mice. Fear conditioning and context-dependent fear memory extinction paradigms were combined with c-Fos imaging and electrophysiological recordings in the dorsal hippocampus (dHip). *Tph2* mutant mice, completely devoid of 5-HT synthesis in brain, displayed accelerated fear memory formation and increased locomotor responses to foot shock. Furthermore, recall of context-dependent fear memory was increased. The behavioral responses were associated with increased c-Fos expression in the dHip and resistance to foot shock-induced impairment of hippocampal long-term potentiation (LTP). In conclusion, increased context-dependent fear memory resulting from brain 5-HT deficiency involves dysfunction of the hippocampal circuitry controlling contextual representation of fear-related behavioral responses.

## Introduction

Anxiety disorders are common and result in substantial economic costs to individuals and society (Bereza et al., 2009). The current global prevalence of anxiety disorders is approximately 7.3%, ranging from 5.4 to 10.4% (Baxter et al., 2013). Many individuals meet diagnostic criteria for multiple anxiety disorders and comorbidity with other psychiatric disorders (Kessler et al., 2005; Miyazaki et al., 2011). A hallmark of anxiety disorders is dysfunctional acquisition and extinction of conditioned fear memories (Grillon, 2002; Milad et al., 2008).

Evidence suggests that serotonin plays an important role in control of anxiety and fear responses (Lesch et al., 1996; Lowry et al., 2005; Maier and Watkins, 2005; Maier et al., 2006; Baratta et al., 2016; Bocchio et al., 2016). Mice with a targeted inactivation of *Tph2* have provided insights into the role of 5-HT in the modulation of anxiety-like behaviors. Previous studies of lifelong deficiency of brain 5-HT synthesis are consistent with the hypothesis that the brain serotonergic system plays an important role in control of anxiety-like behaviors (Mosienko et al., 2015), fear learning, and behavioral responses to stress (Gutknecht et al., 2015), effects that might be due to alterations in GABAergic transmission (Jorgensen et al., 2013; Waider et al., 2013). Furthermore, mice with defects in 5-HT system development leading to reduction of 5-HT neurons showed differential anxiety-like behaviors and fear memory (Hendricks et al., 2003; Dai et al., 2008; Schaefer et al., 2009; Kiyasova et al., 2011; Song et al., 2011; Brooks et al., 2014). Indeed, the 5-HT system is thought to play an essential role in the regulation of fear memory in rodents (Graeff and Zangrossi, 2010; Bocchio et al., 2016). Studies in animals demonstrate a direct anatomical connection between the main sources of serotonin in the brain, the brainstem dorsal and median raphe nuclei as well as forebrain limbic structures, such as the medial prefrontal cortex, hippocampus, and amygdala, that control anxiety and fear responses (Maier et al., 2006; Hale and Lowry, 2011; Fernandez et al., 2016; Muzerelle et al., 2016). Of particular interest to contextual fear conditioning is the dorsal hippocampus (dHip; Bauer, 2015), which receives serotonergic projections primarily from the median raphe nucleus (Azmitia and Whitaker-Azmitia, 1995; McQuade and Sharp, 1997; Lowry, 2002).

Consistent with this hypothesis, acute administration of selective 5-HT reuptake inhibitors (SSRIs) 60 min before testing results in a decrease in contextual fear expression (Hashimoto et al., 1996; Li et al., 2001; Gravius et al., 2006), while it increases conditioned fear expression in auditory fear conditioning setting (Burghardt et al., 2007). Furthermore, peripheral administration of SSRIs decreases neuronal activity, immediate-early gene expression, and plasticity in the hippocampus (Staubli and Otaky, 1994; Igelstrom and Heyward, 2012; Ravinder et al., 2013). In addition to these effects of serotonergic signaling on fear expression, other studies provide support for a role for multiple 5-HT receptor types in the dHip in conditioned fear memory consolidation (Schmidt et al., 2017).

We previously showed that *Tph2* mutant (*Tph2*^-/-^) mice display enhanced acquisition of conditioned fear and escape-oriented behavior in response to aversive foot shock, in association with altered basolateral amygdala function (Waider et al., 2017). Here, we investigated the impact of a lifelong abscence of brain 5-HT synthesis on the contextual domain of fear learning, using fear conditioning combined with an extinction paradigm, functional immunohistochemistry, and electrophysiological recordings within the hippocampal formation.

## Materials and Methods

### Animals

Adult male *Tph2*^+/+^, *Tph2*^+/-^, and *Tph2*^-/-^ mice on a mixed Sv129/C57BL/6N genetic background (Gutknecht et al., 2012), 2–5 months of age, were housed individually in a controlled environment (12 h/12 h light/dark cycle, light phase 7 am–7 pm, 21 ± 0.5°C room temperature, 50 ± 5% humidity) with food and water *ad libitum*. Mice were allowed to acclimate for 1 week before being subjected to fear conditioning experiments. Tests were performed during the light phase between 10:00 and 15:00. All experiments were performed in accordance with the European Parliament and Council Directive (2010/63/EU) and were approved by local authorities (55.2-2531.01-57/12) and (IT: 938/2017-PR).

### Fear Conditioning and Contextual Fear Extinction Training

**FIGURE 1:**
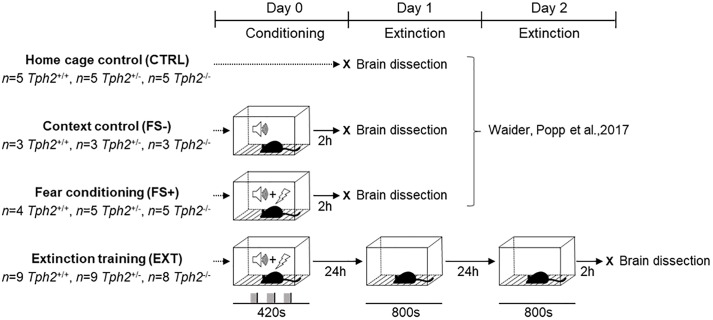
Experimental design for c-Fos immunoreactivity after fear conditioning and context-dependent extinction. *Tph2^+/+^*, *Tph2^+/-^*, and *Tph2^-/-^* mice were exposed to a fear conditioning (FC) protocol and subsequently subjected to a context-dependent extinction protocol. On day 0 mice were placed in the conditioning chamber receiving three pairings of a 20 s tone cue co-terminating with a 2 s foot shock. On day 1 and again on day 2, mice were tested for recall and extinction of context-dependent fear memory using an extinction session consisting of re-exposing the animals to the conditioning context for 800 s without negative reinforcement. Two hours after extinction on day 2, brains were dissected for hippocampal c-Fos expression analysis (EXT) and compared to controls that were left undisturbed in the home cage (CTRL), controls that were exposed to the FC context and tone cue without foot shocks (FS–), and mice that underwent FC with tone-signaled foot shocks (FS+) with brain dissection 2 h after the test (Waider et al., 2017).

In order to assess context-dependent fear memory and extinction, *Tph2*^-/-^, *Tph2*^+/-^, and *Tph2*^+/+^ mice (*n* = 8–9/genotype) were exposed to a fear conditioning protocol as previously described (Waider et al., 2017) and subsequently subjected to a context-dependent extinction protocol (EXT; Figure 1). Briefly, on day 0, mice were placed by a blinded operator in randomized order into the fear conditioning test box (TSE Systems, Homburg, Germany), which was comprised of a transparent Perspex arena (23 cm × 23 cm × 35 cm) on a stainless steel foot shock grid (floor bars 4 mm diameter, distance rod center to rod center 8.9 mm) that was connected to a shocker-scrambler unit for delivering foot shocks of defined duration and intensity (Raab et al., 2018). The arena was placed inside in a square-shaped base frame (outer size: 31 cm × 31 cm) with integrated animal detection sensors (XY and Z axes featuring 16 sensors mounted 14 mm apart). All sensors were scanned with a sampling rate of up to 100 Hz to monitor the animal’s position and movement at high spatial and temporal resolution. The test box was operated in a sound-attenuating housing (52 cm × 52 cm × 65 cm) featuring a loudspeaker and two lamps in the ceiling for software-controlled application of acoustic stimuli and continuous house-light illumination (set to 100 lux in all testing phases), respectively.

Mice were allowed to freely explore for 2 min the test box before receiving three pairings of a 20 s tone cue (80 dB, 4 kHz), co-terminating with a 2 s foot shock (FS, 0.6 mA) at an inter-trial interval (ITI) of 1 min. Mice were removed from the chamber 2 min after the last tone-shock pairing. Starting 24 h after conditioning, mice were tested for recall and extinction of context-dependent fear on two consecutive days (day 1 and 2). Each extinction session consisted of re-exposing the animals to the conditioning context for 800 s without negative reinforcement.

Freezing was defined as no light-beam interruption for at least 2 s and expressed as percentage of time relative to total session duration (Rivero et al., 2015; Raab et al., 2018). Additionally, other indices of locomotor activity and exploratory behavior (i.e., distance moved, mean and maximum velocity, activity vs. inactivity, rearing) were measured continuously by the light-beam detection system. Inactivity was defined as the percentage of time the animal’s speed fell below a threshold of 2 cm/s. Using the same fear conditioning system (TSE FCS 303410 series), Misane et al. (2005) have shown that there is a close linear correlation between operator-scored freezing (using a time-sampling procedure in which the animal was instantly scored as either freezing or active every 10 s) and computer-derived inactivity (activity threshold 1 cm/s) both in context- and tone-dependent memory tests. Moreover, Toth et al. (2012) have shown that there is no difference between hand-scored freezing, TSE-determined freezing (defined as no light-beam interruption for at least 3 s), and Noldus Ethovision-determined inactivity.

To determine acquisition of the conditioned fear response, freezing was measured continuously throughout the training session. The maximum movement velocity was taken as an indicator of the animal’s unconditioned reactivity to foot shock (Waider et al., 2017). During extinction training, freezing was continuously recorded in 20 s time-bins and, except for the first 20 s, averaged into blocks of 1 min to assess recall and extinction of contextual fear memory.

### C-Fos and Parvalbumin Immunostaining in the Dorsal Hippocampus

Two hours after extinction on day 2, the brains of the mice that underwent context-dependent extinction training (EXT) were prepared for immunostaining of parvalbumin (PV) and c-Fos as previously described (Waider et al., 2017). The brain sections were analyzed and compared with the brains of mice that were either left undisturbed in the home cage (CTRL), exposed to the fear conditioning context and tone without foot shocks (FS-), or underwent fear conditioning with tone-signaled foot shocks (FS+) as previously described (Waider et al., 2017; Figure 1). In brief, serially cut 30 μm-thick cryostat sections were used for immunofluorescent stainings. Primary antibodies used were mouse anti-PV (1:200; Swant, Marly, Switzerland) and rabbit anti-c-Fos (1:400; Santa Cruz Biotechnology, Dallas, TX, United States). Sections were incubated in 1:400 diluted secondary antibodies, goat anti-mouse 488 and goat anti-rabbit 555 (Invitrogen, Carlsbad, CA, United States). Pictures were acquired with a motorized inverted system epifluorescence microscope IX81 (Olympus, Tokyo, Japan). Pictures were taken with 20× objective in x-y directions. Images were then processed using CellSense (Olympus, Tokyo, Japan), and corrected for contrast and brightness using ImageJ v2.0.0 (Schindelin et al., 2012). Three to five sections from -1.06 mm bregma to -2.06 mm bregma of the dHip, spaced 180 μm apart, were delineated with contours according to a stereotactic atlas of the mouse brain (Franklin and Paxinos, 1997). Immunoreactive (ir) cells were counted as c-Fos-ir only when the nucleus, according to DAPI (4′, 6-diamidino-2-phenylindole) counterstaining, showed complete fluorescent signal. When the PV signal superimposed or surrounded the cell nucleus, it was counted as c-Fos/PV-ir. The sum of counted cells of all sections per mouse were divided by the total contour area to calculate the cell densities per region of interest.

### Electrophysiology

To investigate the impact of 5-HT deficiency on hippocampal plasticity under basal conditions and after contextual fear conditioning with unsignaled foot shocks, an independent cohort of mice was either left undisturbed (naïve controls) or subjected to a foot shock procedure according to Dai et al. (2008). Mice were placed in the box and allowed to freely explore for 2 min before receiving five foot shocks (0.5 mA, 2 s) with ITI of 2 min. Two minutes after the last foot shock, mice were removed from the chamber and slices were prepared. Field excitatory postsynaptic potentials (fEPSPs) in the CA1 region of the dHip were recorded in transversal slices as previously described (Morini et al., 2011; Mlinar et al., 2015). LTP was induced by theta burst stimulation (TBS) comprised of a single train of 5 bursts of 5 stimuli (100 Hz intra-burst frequency, 5 Hz burst frequency). Stimulation intensity for baseline measurement and LTP induction was set to evoke fEPSP corresponding to 35–40% of the maximal response. Typically, more than one slice was used per mouse and the results of all determinations per genotype and treatment are shown and analyzed in order to account for the overall variability of LTP responses in the different genotypes. Mean values from replicates (2–4) in the same animal were used for genotype x treatment statistical analysis reported in results.

### Statistical Analyses

Data were analyzed using IBM SPSS Statistics 21 (IBM Corp., Armonk, NY, United States) or GraphPad Prism version 6.00 (GraphPad Software, San Diego, CA, United States). Behavioral data were analyzed by two- or three-way mixed analysis of variance (ANOVA) with genotype as the between-subjects factor and with time and day as within-subjects factors. When appropriate, degrees of freedom were corrected using Greenhouse-Geisser estimates of sphericity.

An ANCOVA was performed in addition to the regular ANOVA to adjust for the effect of post-shock freezing (=covariate) on subsequent freezing during extinction sessions. Immunohistochemical and electrophysiological data were analyzed by two-way ANOVA with group and genotype as between-subjects factors. Unless otherwise indicated, Bonferroni *post hoc* tests were performed to evaluate significant main effects or simple effects following a significant interaction. Correlations between behavioral parameters and c-Fos expression were calculated using the Pearson correlation coefficient.

Results are presented as mean ± SEM unless stated otherwise. The significance level was set at *p* < 0.05 and *p* < 0.1 was highlighted as approaching statistical significance.

## Results

### Increased Acquisition of Fear Conditioning in *Tph2*^-/-^ Mice

**FIGURE 2:**
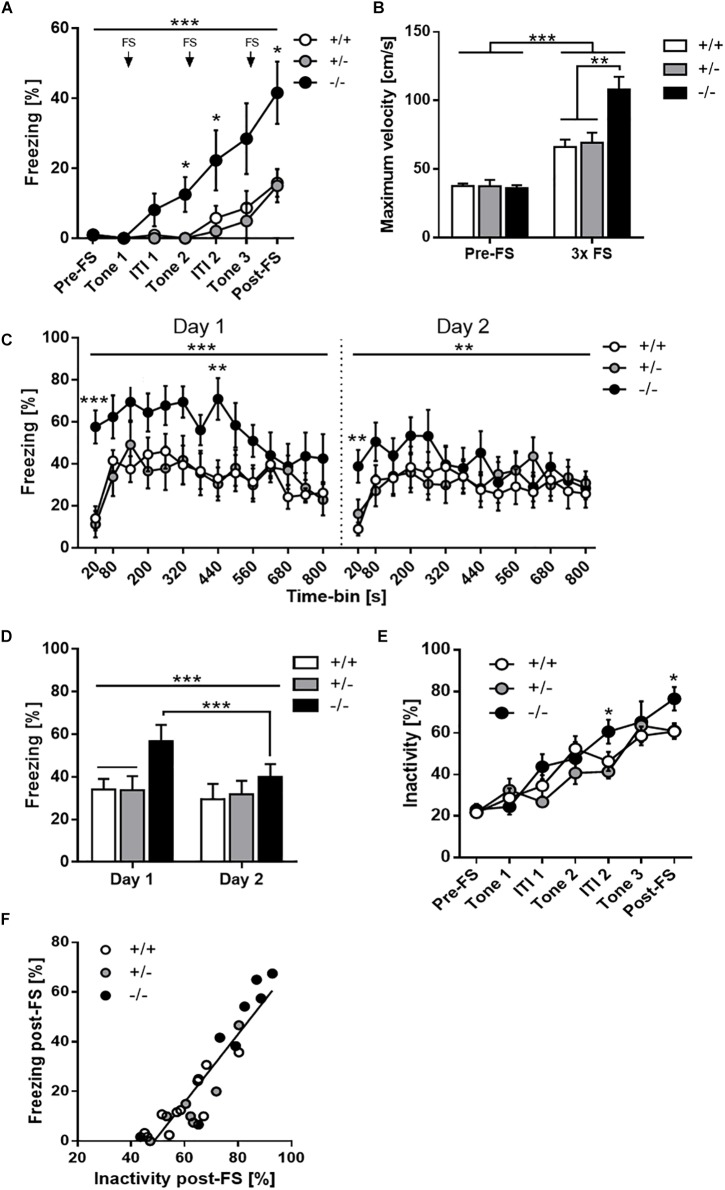
Increased context-dependent fear memory recall but effective contextual extinction learning due to 5-HT deficiency. **(A)** Time-course analysis of the conditioned fear response of *Tph2^+/+^*, *Tph2^+/-^*, and *Tph2^-/-^* mice during acquisition training showing relative freezing levels before the first pairing of a tone cue with a foot shock (pre-FS), during tone presentations and inter-trial intervals (ITI), and after the last foot shock (post-FS). **(B)** Unconditioned foot shock reactivity, measured as maximum movement velocity before the first pairing of a tone cue with a foot shock (pre-FS) and following the foot shock (3 × FS). **(C)** Time-course analysis of the freezing response during contextual fear memory recall and extinction training on day 1 and 2 after conditioning. **(D)** Average freezing scores in the two extinction sessions. **(E)** Time-course analysis of the inactivity level during acquisition training showing relative inactivity levels. **(F)** Positive relationship between freezing scores obtained after the last foot shock (post-FS) in the conditioning session and inactivity scores. Data are shown as means + or ± SEM. ^∗^*p* < 0.05, ^∗∗^*p* < 0.01 and ^∗∗∗^*p* < 0.001.

Two-way mixed ANOVA for freezing during fear acquisition training (Figure 2A) revealed a significant main effect of time [*F*_(2.8,64.4)_ = 17.54, *p* < 0.001] and genotype [*F*_(2,23)_ = 7.70, *p* = 0.003], as well as a time × genotype interaction [*F*_(5.6,64.4)_ = 2.73, *p* = 0.023]. *Post hoc* tests showed that freezing levels were very low before the first tone-shock pairing and gradually increased thereafter over the course of training. However, *Tph2*^-/-^ mice acquired the conditioned fear response more rapidly than *Tph2*^+/-^ and *Tph2*^+/+^ mice, as evidenced by significantly elevated freezing levels *Tph2*^-/-^ mice from the second tone presentation onward. Furthermore, the reactivity to foot shock, as measured by the maximum movement velocity (Vmax), was significantly increased in *Tph2*^-/-^ compared to *Tph2*^+/-^ and *Tph2*^+/+^ mice [genotype effect: *F*_(2,23)_ = 9.63, *p* < 0.001; Figure 2B].

Moreover, comparison of baseline (pre-FS) Vmax and foot shock-induced Vmax revealed a significant increase in all three genotypes (*Tph2*^+/+^: *p* < 0.01, *Tph2*^+/-^: *p* < 0.001 and *Tph2*^-/-^: *p* < 0.0001), thereby demonstrating that *Tph2*^+/+^ and *Tph2*^+/-^ mice showed a clear response to FS, which was further exaggerated in *Tph2*^-/-^ mice [phase x genotype interaction: *F*_(2,23)_ = 8.223, *p* = 0.002].

Besides freezing, we analyzed inactivity using an activity threshold of 2 cm/s. Baseline (pre-FS) inactivity (immobility/resting: ∼20–25%) did not significantly differ between genotypes (*p* = 0.861; Figure 2E). Inactivity increased linearly across tone-FS trials in all three genotypes [main effect of phase: *F*_(4.2,96.2)_ = 55.73, *p* < 0.0001] and reached maximum levels after three tone-FS pairings (∼61% in *Tph2*^+/+^ and *Tph2*^+/-^, ∼76% in *Tph2*^-/-^ mice). Similar to freezing, *Tph2*^-/-^ mice were significantly less active than *Tph2*^+/-^ and *Tph*^+/+^ mice after the 2nd and 3rd tone-FS pairing [phase x genotype interaction: *F*_(8.4,96.2)_ = 2.27, *p* = 0.026]. Moreover, there was a close linear correlation between post-shock freezing and post-shock inactivity (*r* = 0.926, *p* < 0.0001) with similar steepness of regression slopes in *Tph2*^+/+^, *Tph2*^+/-^ and *Tph2*^-/-^ mice (Figure 2F).

### Increased Recall but Effective Extinction of Contextual Fear Memory in *Tph2*^-/-^ Mice

Analysis of freezing during extinction training on day 1 and 2 after fear conditioning revealed a significant time-bin x genotype [*F*_(9.5,149.5)_ = 2.02, *p* = 0.041, Figure 2C] and day × genotype [*F*_(2,299)_ = 5.29, *p* = 0.013, Figure 2D] interaction, but no genotype main effect [*F*_(2,23)_ = 2.09, *p* = 0.147]. *Post hoc* tests showed that, within sessions, *Tph2*^-/-^ mice promptly froze upon placement into the conditioned context, while freezing onset was slightly delayed in *Tph2*^+/-^ and *Tph2*^+/+^ mice. Moreover, freezing levels of *Tph2*^-/-^ mice remained elevated during the first minutes of testing but were indistinguishable from the other genotypes at the end of the session due to a steeper decline of the freezing response in *Tph2*^-/-^ compared to *Tph2*^+/-^ and *Tph2*^+/+^ mice (Figure 2C). Accordingly, *Tph2*^-/-^ mice showed a significantly stronger decrease of freezing than *Tph2*^+/-^ and *Tph2*^+/+^ mice across the two extinction sessions (Figure 2D).

Since *Tph2*^-/-^ mice displayed significantly enhanced acquisition of conditioned fear and post-shock freezing levels in the conditioning session, we performed an analysis of covariance to control for differences in post-shock freezing levels. Three-way mixed ANCOVA confirmed a significant effect of post-shock freezing [*F*_(1,22)_ = 12.56, *p* = 0.002] as well as a time-bin x genotype [*F*_(9.9,138.6)_ = 2.46, *p* = 0.011] and day x genotype [*F*_(2,286)_ = 2.73, *p* = 0.087] interaction on the freezing scores obtained during extinction training. *Post hoc* tests again showed that *Tph2*^-/-^ mice froze significantly more than *Tph2*^+/-^ and *Tph2*^+/+^ mice during the first 20 s of context re-exposure. However, freezing levels were indistinguishable among genotypes thereafter. Furthermore, *Tph2*^-/-^ mice were able to extinguish the conditioned fear more efficiently than *Tph2*^+/-^ and *Tph2*^+/+^ mice, as evidenced by a stronger decline of the freezing response both within and across the two extinction sessions.

Taken together, these results indicate that 5-HT deficiency due to *Tph2* inactivation augmented context-dependent fear memory recall and facilitated both intra- and intersession extinction learning.

### Increased c-Fos Activation Due to Fear Conditioning in *Tph2*^-/-^ Mice

**FIGURE 3:**
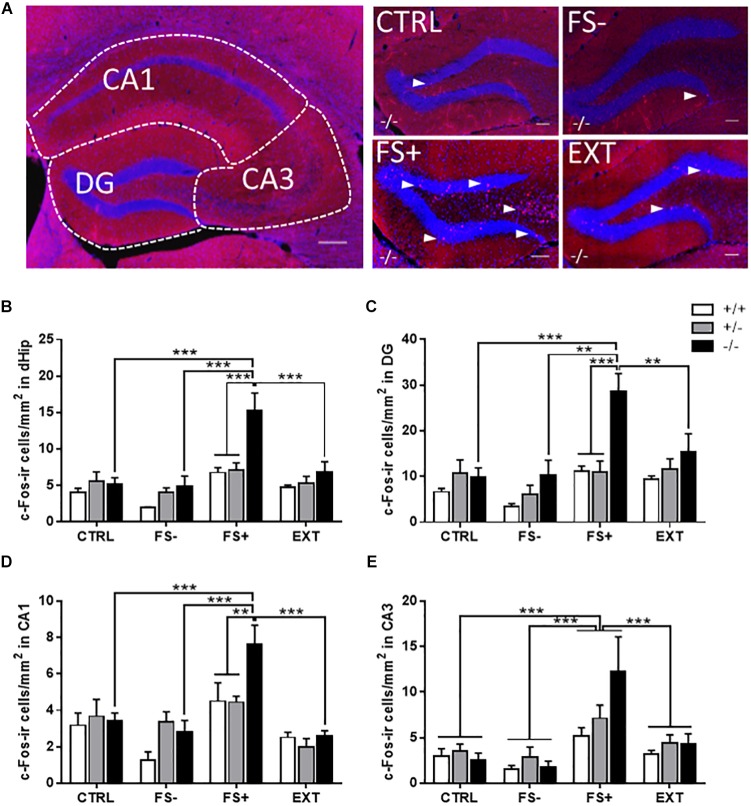
Increased c-Fos activity in the dorsal hippocampus (dHip) of *Tph2^-/-^* mice following fear conditioning. (**A**, left panel) Overview of the dHip indicating the subregions dentate gyrus (DG), cornu ammonis area 1 (CA1), and cornu ammonis area 3 (CA3) and (**A**, right panel) higher magnification images depicting the DG of representative *Tph2^-/-^* mice for the different groups: home cage controls (CTRL), context controls subjected to the conditioning procedure without foot shocks (FS–), fear-conditioned mice receiving three tone-signaled foot shocks (FS+), fear-conditioned mice that underwent two extinction training sessions (EXT). Quantification of c-Fos-ir cell densities in **(B)** the total dHip, **(C)** DG, **(D)** CA1, and **(E)** CA3 of *Tph2^+/+^*, *Tph2^+/-^*, and *Tph2^-/-^* mice of the four different groups. Arrowheads in **(A)** indicate c-Fos-ir cells (red), DAPI-stained cell nuclei (blue). Scale bars in **(A)**: 200 μm (left panel) and 100 μm (right panel). Data are shown as means + SEM. ^∗∗^*p* < 0.01; ^∗∗∗^*p* < 0.001.

Because of the strong effect of 5-HT deficiency on post-shock freezing, an indicator of short-term memory for contextual fear (Fanselow, 1980), we analyzed c-Fos activation in the dHip and its subregions, dentate gyrus (DG), cornu ammonis area 1 (CA1), and 3 (CA3) of *Tph2*^-^*^/^*^-^, *Tph2^+/^*^-^ and *Tph2^+/+^* mice that were either left undisturbed in the home cage (CTRL), exposed to the fear conditioning procedure without foot shocks (FS-) or with foot shocks (FS+), or subjected to fear conditioning and subsequent extinction training (EXT) (Figure 3).

An increased density of c-Fos-ir cells was found in the dHip, especially in the granule cell layer and hilus of the DG (Figure 3A) of FS+ *Tph2*^-/-^ mice. Quantification confirmed a significant genotype x group interaction for the density of c-Fos-ir cells in the total dHip [*F*_(6,51)_ = 3.05, *p* = 0.0126; Figure 3B], in the DG [*F*_(6,51)_ = 2.31, *p* = 0.0477; Figure 3C], and in the CA1 area [*F*_(6,51)_ = 2.59, *p* = 0.0288; Figure 3D]. *Post hoc* analyses showed that the number of c-Fos-ir cells was increased in the total dHip, DG and CA1 of *Tph2*^-/-^ after fear conditioning relative to respective *Tph2*^+/-^ and *Tph2*^+/+^ FS+ controls as well as to CTRL, FS-, and EXT *Tph2*^-/-^ mice (*p* < 0.01). A similar activation pattern was observed in the CA3 region, although only a group main effect was detected [*F*_(3,51)_ = 9.59, *p* < 0.0001; Figure 3E], with a significantly increased density of c-Fos-ir cells in FS+ animals compared to CTRL, FS-, and EXT mice (all *p* < 0.001).

Correlation analysis of the conditioned fear response (post-shock freezing) with the c-Fos-ir cell density in FS- and FS+ animals detected a strong positive relationship specifically in *Tph2*^-/-^ mice (*r* ≥ 0.7501, *p* ≤ 0.0321; Table 1). A similar but less exclusive correlation pattern was observed for the density of c-Fos-ir cells with the unconditional response to FS (shock reactivity). Taken together, these data indicate that fear conditioning in *Tph2*^-/-^ mice increased hippocampal activity, while context-dependent extinction training of fear memory normalized hippocampal c-Fos expression within the dHip of *Tph2*^-/-^ mice.

**Table 1:** Pearson’s correlation of shock reactivity and post-shock freezing with c-Fos density in the dorsal hippocampus and its subregions of FS- and FS+ animals.

		Shock reactivity				Post-shock freezing			
		Total	+/+	+/–	–/–	Total	+/+	+/–	–/–
*dHip*	*r*	0.6636	0.7071	0.5517	0.7493	0.6193	0.2035	0.2821	0.8590
	*p*	0.0006	0.0756	0.1563	0.0324	0.0016	0.6616	0.4985	0.0063
*DG*	*r*	0.6592	0.7065	0.3972	0.8409	0.6083	0.2151	0.1633	0.9279
	*p*	0.0006	0.0759	0.3298	0.0089	0.0021	0.6432	0.6992	0.0009
*CA1*	*r*	0.6453	0.4586	0.8015	0.7217	0.5401	–0.0100	0.6363	0.7501
	*p*	0.0009	0.3006	0.0168	0.0433	0.0078	0.9830	0.0898	0.0321
*CA3*	*r*	0.5561	0.7337	0.4562	0.5655	0.5950	0.3135	0.2460	0.7541
	*p*	0.0059	0.0605	0.2558	0.1441	0.0027	0.4936	0.5570	0.0307


### Foot Shock Reduces C-Fos Activation of PV-ir Cells in 5-HT-Deficient Mice

**FIGURE 4:**
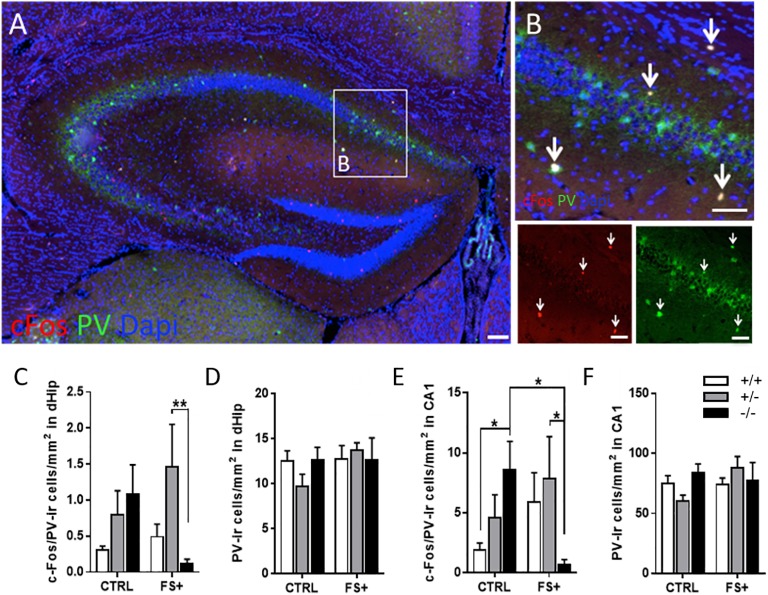
Lifelong brain 5-HT synthesis deficiency prevents activation of parvalbumin-ir neurons in fear conditioning. **(A)** Anti-parvalbumin (PV) (green) and c-Fos (red) immunofluorescent staining with DAPI in the dorsal hippocampus (dHip) of a fear conditioned *Tph2^+/-^* mouse. **(B)** Magnified with magnified area of cornu ammonis area 1 (CA1). Density of c-Fos/PV-ir cells of home cage control (CTRL) and fear-conditioned (FS+) *Tph2^+/+^*, *Tph2^+/-^*, and *Tph2^-/-^* mice in **(C)** dHip and **(E)** CA1. PV-ir cell density of CTRL and FS+ *Tph2^+/+^*, *Tph2^+/-^*, and *Tph2^-/-^* mice in **(D)** dHip and **(F)** CA1. Data are shown as means + SEM. ^∗^*p* < 0.05; ^∗∗^*p* < 0.01. Two-way ANOVA followed by Fisher’s LSD *post hoc* test. Scale bar: 100 μm.

Since PV-ir neurons in the hippocampus were previously shown to be involved in contextual memory (Donato et al., 2013), we analyzed c-Fos-ir neurons in the dHip by double-immunofluorescent staining with PV, a marker of a subset of inhibitory GABAergic interneurons (Gulyas et al., 1999). Because c-Fos-ir densities did not differ between CTRL and FS- animals but were highly increased in fear-conditioned FS+ *Tph2*^-/-^ mice, we focused our further analyses on the FS+ group compared to the CTRL group (Figure 4). PV-ir cells were predominantly found in the CA3 and CA1 regions of the dHip, specifically in the stratum lacunosum, pyramidal layer, and stratum oriens with a high density of c-Fos/PV double-ir neurons in the CA1 region (Figure 4B,E). In the total dHip, ANOVA detected an almost significant genotype x group interaction for the density of c-Fos/PV double-ir neurons [*F*_(2,24)_ = 3.14, *p* = 0.06; Figure 4C]. PV-ir neurons in FS+ *Tph2*^-/-^ mice showed reduced c-Fos-ir cells relative to CTRL *Tph2*^-/-^ mice (*p* = 0.053), while FS+ *Tph2*^+/+^ and *Tph2*^+/-^ mice displayed no alterations compared to respective CTRL mice. Within the FS+ group, *Tph2*^+/-^ mice showed the highest c-Fos/PV-ir density compared to *Tph2*^+/+^ (*p* = 0.05) and *Tph2*^-/-^ (*p* = 0.009) mice.

In CA1, a similar but significant genotype x group interaction was detected for the density of c-Fos/PV double-ir neurons [*F*_(2,24)_ = 4.77, *p* = 0.018; Figure 4E]. *Post hoc* tests revealed that CTRL *Tph2*^-/-^ mice showed increased c-Fos/PV double-ir cell densities relative to CTRL *Tph2*^+/+^ (*p* = 0.047). However, FS+ *Tph2*^-/-^ mice showed reduced c-Fos/PV double-ir cell densities relative to CTRL *Tph2*^-/-^ as well as FS+ *Tph2*^+/-^ (*p* < 0.05) and FS+ *Tph2*^+/+^ (*p* < 0.1) mice. Neither differences among genotypes nor among CTRL and FS+ groups were found for the density of PV-ir neurons in dHip (Figure 4D) and CA1 (Figure 4F). Altogether these data indicate increased recruitment of PV neurons in the dorsal CA1 region of CTRL *Tph2*^-/-^ mice, which is absent after fear conditioning.

### LTP Impairment by Inescapable Foot Shock Is Absent in *Tph2*-Deficient Mice

**FIGURE 5:**
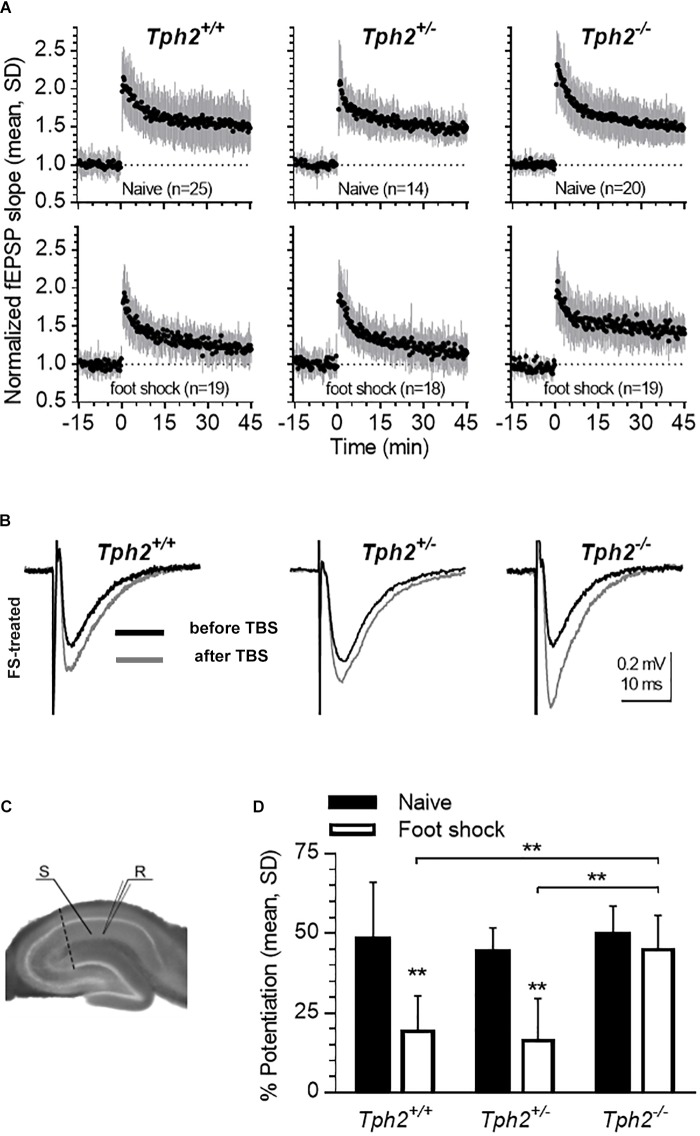
Lack of 5-HT impedes foot shock-induced reduction in hippocampal long-term potentiation (LTP). **(A)** Time-course of LTP in slices obtained from naïve and foot shock-treated mice (*Tph2^+/+^*, *Tph2^+/-^*, and *Tph2^-/-^* mice; naïve: *n* = 8,6,5; foot shock: *n* = 5,6,6). Mean ± SD of fEPSP slope values normalized to the baseline mean from 5 to 0 min before theta burst stimulation (TBS). Numbers (n) in graphs indicate slices tested, including replicates per mouse. **(B)** Representative traces of recordings foot shock-treated *Tph2^+/+^*, *Tph2^+/-^*, and *Tph2^-/-^* mice before and after TBS. **(C)** Positioning of stimulation electrode (S) on Schaffer collaterals and recording electrode (R) in CA1. The dashed line indicates a cut made between cornu ammonis area 1 (CA1) and cornu ammonis area 3 (CA3) to prevent recurrent propagation of action potentials. **(D)** LTP is expressed as the percent change measured 40–45 min after TBS in respect to baseline between naïve and foot shock-treated *Tph2^+/+^*, *Tph2^+/-^*, and *Tph2^-/-^* mice. ^∗∗^*p* < 0.01. Two-way ANOVA, followed by Sidak’s *post hoc* test.

In the CA1 region, plasticity is modulated by endogenous 5-HT (Mlinar et al., 2015). Here, we investigated the impact of 5-HT deficiency on TBS-induced LTP of fEPSP in the hippocampal CA1 region of *Tph2*-deficient mice (Figure 5C). Similar LTP of fEPSP responses were found across genotypes in naïve mice (Figure 5A, upper panels), indicating that basic mechanisms underlying LTP are preserved in the life-long absence of 5-HT. Since the 5-HT system has been implicated in foot shock-induced impairment of LTP (Dai et al., 2008), we compared LTP in slices obtained from animals exposed to repeated foot shock stress (Figure 5A, lower panels, B,D). Two-way ANOVA revealed a significant genotype x group interaction [*F*_(2,30)_ = 9.37 *p* = 0.0484; Figure 5D]. *Post hoc* analysis confirmed decreased LTP in *Tph2^+/+^* (*p* = 0.0011) and *Tph2^+/^*^-^ (*p* = 0.002) mice after foot shock relative to naïve controls, an effect that was absent in *Tph2*^-/-^ mice (Figure 5A,B,D) indicating that presence of 5-HT during foot shock is required for the stress-induced impairment of LTP.

## Discussion

We previously showed that lifelong absence in brain 5-HT synthesis following constitutive *Tph2* inactivation in mice enhanced acquisition of conditioned fear and promoted unconditioned escape responses to electric foot shock (Waider et al., 2017). Here, we were able to replicate and extend these findings, which indicate that constitutive *Tph2* inactivation results in faster acquisition of conditioned fear and increased escape-like behavior in response to foot shock, as well as enhanced contextual representation of the fear memory, but functional extinction of the context. By combining fear conditioning with immediate-early gene expression, our data emphasize that lifelong 5-HT deficiency renders the dHip hyperexcitable in fear conditioning.

The dHip encodes the contextual component of fear learning during early acquisition phases (Fanselow and Dong, 2010; Maren et al., 2013). Furthermore, direct glutamatergic projections from the BLA to the dHip have been shown to be both necessary and sufficient for repeated long-term foot shock-mediated memory impairments (Rei et al., 2015), while neurons in the BLA become activated during hippocampal theta network activity or optogenetic stimulation of CA1 pyramidal neurons (Bienvenu et al., 2012; Bazelot et al., 2015).

Because compensation through a 5-HT-dependent mechanism are absent in *Tph2*^-/-^ mice (Gutknecht et al., 2012; Waider et al., 2017), resilience to foot shock-mediated memory impairments may be derived directly from absence of 5-HT signaling in the dHip. Indeed, inescapable foot shock and novel aversive contexts were reported to impair contextual memory and the induction of LTP in the hippocampal CA1 region (Foy et al., 1987; Sacchetti et al., 2002; Dai et al., 2008).

Moreover, it has long been suggested that 5-HT counteracts the consolidation of stressful memories, presumably mediated by 5-HT1A receptors in the dHip, which then may lead to tolerance with chronic aversive events (Graeff et al., 1996). In contrast to *Lmx1b* cKO mice, which lack raphe 5-HT neurons (Dai et al., 2008), 5-HT neurons in *Tph2*^-/-^ mice are unable to synthesize 5-HT, but are functionally preserved (Gutknecht et al., 2012; Montalbano et al., 2015).

Here, we chose a consolidated foot shock protocol (Dai et al., 2008) to investigate whether the absence of 5-HT influences hippocampal LTP formation to allow direct comparison of our data with those obtained using a different animal model of 5-HT system impairment. Although the protocol applied for the LTP and the behavioral experiments differed and, therefore, quantitative correlation of plasticity impairment with the behavioral effects was not possible, our results demonstrate that 5-HT is required for foot shock-induced impairment of LTP. Indeed, 5-HT has been shown to shift hippocampal activity along the longitudinal axis toward the ventral part (Mlinar and Corradetti, 2018). Thus, an absence of this mechanism in *Tph2*^-/-^ mice may result in an overactivation of the hippocampal circuitry, manifested as enhanced c-Fos immunostaining and overrepresentation of the contextual component in fear learning. In line with this, a mouse model lacking hippocampal serotonergic input showed alterations in contextual fear memory with no differences in LTP formation under baseline conditions (Fernandez et al., 2017).

However, recall of context-dependent aversive memory is exaggerated in *Tph2*^-/-^ mice, supporting the view that 5-HT mediates inhibition of context-dependent aversive memories through inhibition of LTP, while under non-aversive conditions, increased endogenous 5-HT release facilitated LTP induction in the CA1 region, and may underlie the *in vivo* positive effects of augmented 5-HT tone on cognitive performance (Mlinar et al., 2015).

In this respect, it may be of interest in a follow up study to analyze the response of the ventral hippocampus, which is involved in emotional fear processing (Fanselow and Dong, 2010), and is modulated differentially by 5-HT along its longitudinal axis (Mlinar and Corradetti, 2018).

Formation of context-dependent memories requires changes in the expression of calcium-binding proteins of GABAergic interneurons, including hippocampal PV-ir cells (Bienvenu et al., 2012; Donato et al., 2013, 2015). In line with previous studies, 5-HT has been shown to modulate PV-specific neurons in the hippocampus (Gulyas et al., 1999). Our results hint toward an involvement of CA1 PV-ir neurons in context-dependent fear conditioning, which is prevented in *Tph2*^-/-^ mice, although activity of the hippocampus is generally increased. Especially in *Tph2^+/^*^-^ mice, fear conditioning recruited the highest number of PV-ir neurons. Thus, it seems that 5-HT-dependent activation of PV neurons may protect the dHip from overactivation. Furthermore, the increased hippocampal activity in *Tph2*^-/-^ mice due to fear conditioning may prevent an increased response of the BLA, as observed in *Tph2^+/-^* mice, to inhibit flight or panic responses (Waider et al., 2017). Thus, further studies are required to investigate the differential role of 5-HT on PV neuron-dependent theta synchronization in Hip and BLA (Amilhon et al., 2015).

Increased active coping in novel, aversive, and inescapable situations is mediated through an amygdala-ventrolateral periaqueductal gray (vlPAG) circuit, mediated through the dorsolateral PAG (Hale et al., 2012; Tovote et al., 2016). 5-HT neurons of the vlPAG inhibit panic-like responses mediated by dorsolateral PAG neurons (Pobbe et al., 2011; Spannuth et al., 2011). Indeed, *Tph2^-/-^* mice show increased post-shock freezing and increased flight responses, although the BLA did not respond to foot shock with an altered activity compared to *Tph2^+/+^* mice (Waider et al., 2017). Thus, the increased hippocampal response in mice completely devoid of 5-HT-mediated regulatory mechanisms may influence the PAG in its role to translate inputs from the amygdala into an appropriate behavioral response, resulting in increased freezing and flight behaviors.

In conclusion, exaggerated context-dependent fear memory and shock reactivity resulting from brain 5-HT deficiency likely involves dysfunction of the raphe-hippocampal innervation controlling fear-related behavioral responses and is presumably due to the failure of 5-HT receptor-mediated inhibition of hippocampal circuitries. Furthermore, our data indicate largely effective extinction learning, during repetitive context exposure without negative reinforcement and without 5-HT functioning. Thus, context-dependent extinction training may represent a strategy to adapt behavioral therapy for patients suffering from 5-HT system dysfunction associated with anxiety-, stress-, and trauma-related disorders.

## Author Contributions

JW, RC, ASB, and KPL designed and supervised the study. JW, SP, BM, AM, FB, BA, ET, RK, CT, NA, ES, and ASB performed and analyzed the experiments. JW, SP, BM, RC, CL, and KPL interpreted the results and wrote the manuscript.

## Conflict of Interest Statement

The authors declare that the research was conducted in the absence of any commercial or financial relationships that could be construed as a potential conflict of interest.
